# FoundationOne CDx testing accurately determines whole arm 1p19q codeletion status in gliomas

**DOI:** 10.1093/noajnl/vdab017

**Published:** 2021-02-04

**Authors:** Radwa Sharaf, Dean C Pavlick, Garrett M Frampton, Maureen Cooper, Jacqueline Jenkins, Natalie Danziger, James Haberberger, Brian M Alexander, Timothy Cloughesy, William H Yong, Linda M Liau, Phioanh L Nghiemphu, Matthew Ji, Albert Lai, Shakti H Ramkissoon, Lee A Albacker

**Affiliations:** 1 Foundation Medicine, Inc., Cambridge, Massachusetts and Morrisville, North Carolina; 2 Department of Neurology, David Geffen School of Medicine, University of California Los Angeles, Los Angeles, California; 3 Department of Pathology and Laboratory Medicine, David Geffen School of Medicine, University of California Los Angeles, Los Angeles, California; 4 Department of Neurosurgery, David Geffen School of Medicine, University of California Los Angeles, Los Angeles, California; 5 Wake Forest Comprehensive Cancer Center and Department of Pathology, Wake Forest School of Medicine, Winston-Salem, North Carolina

**Keywords:** CGP, comprehensive genomic profiling, FoundationOne, F1CDx, glioma, 1p19q

## Abstract

**Background:**

Molecular profiling of gliomas is vital to ensure diagnostic accuracy, inform prognosis, and identify clinical trial options for primary and recurrent tumors. This study aimed to determine the accuracy of reporting the whole arm 1p19q codeletion status from the FoundationOne platform.

**Methods:**

Testing was performed on glioma samples as part of clinical care and analyzed up to 395 cancer-associated genes (including *IDH1/2*). The whole arm 1p19q codeletion status was predicted from the same assay using a custom research-use only algorithm, which was validated using 463 glioma samples with available fluorescence in-situ hybridization (FISH) data. For 519 patients with available outcomes data, progression-free and overall survival were assessed based on whole arm 1p19q codeletion status derived from sequencing data.

**Results:**

Concordance between 1p19q status based on FISH and our algorithm was 96.7% (449/463) with a positive predictive value (PPV) of 100% and a positive percent agreement (PPA) of 91.0%. All discordant samples were positive for codeletion by FISH and harbored genomic alterations inconsistent with oligodendrogliomas. Median overall survival was 168 months for the *IDH1/2* mutant, codeleted group, and 122 months for *IDH1/2* mutant-only (hazard ratio (HR): 0.42; *P* < .05).

**Conclusions:**

1p19q codeletion status derived from FoundationOne testing is highly concordant with FISH results. Genomic profiling may be a reliable substitute for traditional FISH testing while also providing *IDH1/2* status.

**Key Points**

- F1CDx can accurately determine the 1p19q status of gliomas with a concordance of 96.7% against FISH.
- F1CDx testing may be used in preference to FISH.
- F1CDx detects many of the relevant molecular biomarkers for gliomas in one test.

**Importance of the Study**

The integration of genomic biomarkers into brain tumor classification has improved the diagnostic accuracy and led to the development of molecularly stratified clinical trials. Particularly, whole arm 1p19q codeletion status is a valuable diagnostic, prognostic and predictive biomarker in gliomas and has traditionally been performed by FISH. However, FISH is unable to differentiate between whole chromosome arm deletions and smaller focal deletions. This distinction is important given the association of 1p19q whole arm codeletion with improved survival. In this work, we show that comprehensive genomic profiling using F1 or F1CDx testing can accurately detect whole arm loss of 1p and/or 19q, in addition to providing information about genomic alterations (eg *IDH1/2*, *pTERT*, *TP53*) within the sample. Thus, F1 or F1CDx testing may be used in preference to FISH, given their ability to detect many of the relevant molecular biomarkers for gliomas in one test with improved accuracy.

Gliomas are the most common malignant primary brain tumors,^1,2^ comprising 26% of all CNS tumors and 81% of malignant tumors. For these patients, outcomes remain poor, with a 2-year survival rate of only 2% in patients greater than 65 years old and 30% in patients under 45 years old.^3^ Historically, histopathological criteria alone separated gliomas into diagnostic categories. However, recent updates to the WHO Classification of CNS tumors have emphasized genomic biomarkers, particularly *IDH1/2*, 1p19q co-deletion, and *ATRX*. This integrated diagnostic workflow has generated the need for more precise diagnostic entities, which stands to improve the clinical outcomes of patients.^4^

*IDH1/2* mutated gliomas account for approximately 20% of all glioma samples.^5^ Oligodendrogliomas are characterized by an *IDH1/2* mutation and whole arm 1p19q co-deletion, while adult lower grade (grade II and III) diffuse astrocytomas frequently harbor an *IDH1/2* mutation but lack 1p19q codeletion.^4,6–8^ Prognostically, the presence of these two markers, characteristic of oligodendrogliomas, confers a favorable prognosis.^9–18^ A study of 149 WHO grade II glioma samples reported that *IDH1/2*-mutated, 1p19q codeleted gliomas showed longer overall survival (OS) compared to other molecular subtypes (*P* < .05).^19^ Comparisons of histological subtypes were unable to significantly stratify patients by outcomes (*P* = .16), emphasizing the value of molecular stratification of diffuse gliomas over traditional histologic strategies.^7,19^

Typically, the *IDH1/2* mutation status and 1p19q codeletion status are identified through a combination of FISH to test for 1p19q codeletion and sequencing or immunohistochemistry (IHC) to detect mutations in *IDH1/2*. Notably, IHC testing is specific to only the *IDH1* R132H protein isoform which accounts for approximately 80–85% of all *IDH1/2* mutations.^20^ With regards to FISH, many commercially available results are reported based on a “minimally deleted region” approach.^21^ FISH probes are sensitive but not specific for the detection of 1p19q codeletion because FISH is unable to distinguish loss of the whole chromosome arm from a focal deletion. This distinction is important because overall survival is inferior in those with focal as opposed to whole arm 1p19q codeletion.^18,22^

In this study, we sought to assess the accuracy of whole arm 1p19q codeletion calling using F1 or F1CDx in gliomas. We tested our algorithm using samples from 463 glioma samples that were sequenced clinically using F1 or F1CDx and compared the predicted codeletion status to that from FISH. Finally, we analyzed clinical outcomes grouped by their *IDH1/2* and F1/F1CDx-derived whole arm 1p19q codeletion status.

## Methods

F1/F1CDx testing was performed as part of routine clinical care in a College of American Pathologists (CAP)-accredited, Clinical Laboratory Improvement Amendments (CLIA)-certified, New York State-regulated reference laboratory (Foundation Medicine, Inc). All samples underwent central histopathologic review by a board-certified neuropathologist (S.H.R.) using World Health Organization criteria. This study was approved by the Western Institutional Review Board (IRB# 20152817) and includes a waiver of informed consent and a HIPAA waiver of authorization.

At least 50 ng of DNA per specimen was extracted from formalin-fixed paraffin-embedded samples from patients with brain tumors. Next-generation sequencing (NGS) was performed using hybridization-captured, adaptor ligation-based libraries to high, uniform coverage in up to 395 cancer-related genes and the intronic regions of 28 genes commonly involved with rearrangement mutations. Glioma samples were sequenced using one of two assays, F1 or F1CDx, thus results were analyzed separately for each assay. The two assays differ in the baitsets used for hybridization capture to enrich for cancer-related genes. Throughout the manuscript, gene alterations are discussed only if the gene is baited on both baitsets.

Sequence data were analyzed for clinically relevant classes of genomic alterations, defined as alterations that are targetable by anticancer drugs currently available on the market or in registered clinical trials. These alterations include base-pair substitutions, insertions/deletions, copy number alterations, rearrangements/fusions. Tumor mutational burden (TMB) was calculated from ~1 MB of the sequenced genome; patients with TMB > 8.7 mutations per megabase were considered “hypermutated”.^23^

We ran a custom research-use only algorithm to assess the whole arm 1p19q codeletion from sequencing data of 463 (162 F1, 148 F1CDx, and 153 UCLA sequenced on F1 or F1CDx) glioma samples. A copy number modeling algorithm utilized the coverage data of baited regions of the genome within each sample, normalized to a process-matched control, to model the copy number of each segment. The minor allele frequencies of up to 59,622 single nucleotide polymorphisms (SNPs) distributed across each segment were used to determine the loss of heterozygosity (LOH) status of each segment. The algorithm then calculated the percentage of the 1p and 19q arms that were monoallelic (under LOH).^24^ FISH tests for 1p19q codeletion were performed by the submitting institutions and results were abstracted from clinical pathology reports. Concordance of the 1p19q status from F1/F1CDx vs. FISH was calculated.

Clinical outcomes were assessed for 519 neuro-oncology patients seen at UCLA who received Foundation Medicine’s genomic profiling between August 2012 and March 2019. Patients were included whether FISH 1p19q testing was performed or not. There is an overlap of 143 patients between the 519 UCLA samples with available clinical outcomes data and the 153 samples used for the comparison against FISH. All patients provided informed consent under a UCLA Institutional Review Board-approved protocol. OS was defined as the time between the date of initial diagnosis and the date of censor/death. Progression-free survival (PFS) was defined as the time between the date of initial diagnosis and the date of tumor progression following standard of care treatment. The response assessment in neuro-oncology (RANO) criteria was used by treating clinicians to determine tumor progression.

## Results

### Patient Cohort

Our validation study comprised 463 glioma samples (162 from F1 testing, 148 F1CDx testing, and 153 from UCLA) with available FISH results (Table 1). Unlike the UCLA cohort, samples within the F1 and F1CDx cohort underwent primary selection based on availability of 1p19q FISH status in the submitted pathology report. The median age at testing was 44 years. Tumor types analyzed in this study include oligodendroglioma, oligoastrocytoma, astrocytoma, glioblastoma, glioma (not otherwise specified, NOS) as well as rare ependymoma, medulloblastoma, and low-grade glioma/glioneuronal tumors (Table 1).

**Table 1.** Overview of samples included in the study

	Total samples [N = 463]
Male gender, N [%]	266 [57.5%]
Age, median [Q1:Q3]	44 [33–57]
Tumor purity, median [Q1:Q3]	40% [30%–60%]
*IDH1/2* mutated samples, N [%]	281 [60.7%]
*TP53* mutated samples, N [%]	212 [45.8%]
*ATRX* mutated samples, N [%]	120 [25.9%]
*pTERT* mutated samples, N [%]	245 [52.9%]
Diagnoses:	
Oligodendroglioma, N [%]	160 [34.6%]
Astrocytoma, N [%]	108 [23.3%]
Oligoastrocytoma, N [%]	47 [10.2%]
Glioblastoma, N [%]	99 [21.4%]
Glioma (NOS), N [%]	44 [9.5%]
Low-grade gliomas/glioneuronal tumors, N [%]	3 [<1%]
Ependymoma, N [%]	1 [<1%]
Medulloblastoma, N [%]	1 [<1%]

### The Landscape of Gene Mutations Associated with Diffuse Gliomas

**Figure 1.**
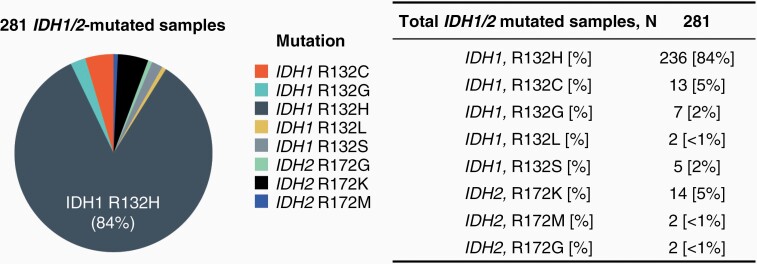
Pie chart depicting the distribution of *IDH1/2* mutations across samples. Results are summarized in the table.

Our cohorts were enriched for *IDH1/2* positivity, where *IDH1/2* mutations with known pathogenicity were present in 60.7% (281/463) of samples with available FISH testing results (Table 1). Particularly, *IDH1* R132H was the most frequent mutation identified, comprising 84% (236/281) of *IDH1/2*-mutated gliomas (Figure 1). Small populations of *IDH2*-mutated gliomas were identified as well, with 6% (18/281) of *IDH1/2*-mutated gliomas harboring *IDH2* R172 mutations (Figure 1 and Table 1). For *IDH1/2*-wild type (WT), the median age at testing was 55 years and for *IDH1/2*-mutated, the median age at testing was 40 years.

The incidence of pathogenic *TP53* alterations was investigated across our cohort and were present in 46% (212/463) of all samples; while alterations in *ATRX* and *pTERT* were present in 26% (120/463) and 53% (245/463) of all glioma samples, respectively.

### Predicting Whole Arm 1p19q Codeletion Status from Foundation Testing

**Figure 2.**
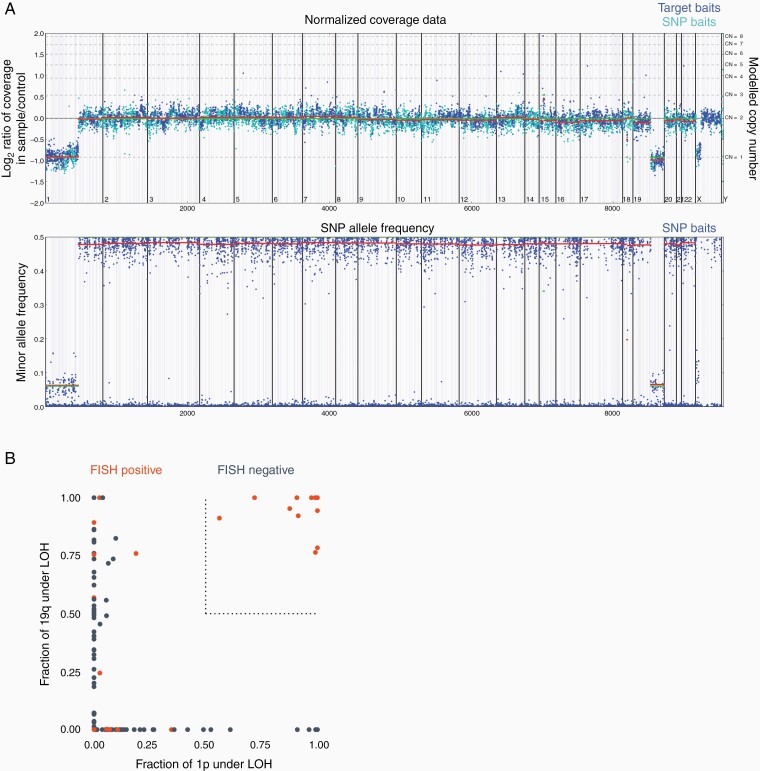
(A) Top plot shows the normalized coverage data for a sample with 1p19q codeletion, normalized to a process-matched control. Blue dots represent target baits and cyan dots represent SNP baits. The bottom plot shows the minor allele frequency for SNPs found within this patient. (B) Fraction of chr1p (x-axis) and chr19q (y-axis) under LOH across all 463 samples. Samples that were categorized as positive for codeletion by FISH are shown in orange and those categorized as negative are shown in dark blue. The cutoff of 0.5 used for our computational 1p19q codeletion algorithm is shown as a dotted line. All samples called as positive by our testing were FISH positive, depicted in orange. Discordant samples are depicted in orange (FISH positive) but lie outside of our dotted cut-off line for the fraction of chr1p and chr19q that is under LOH.

**Figure 3.**
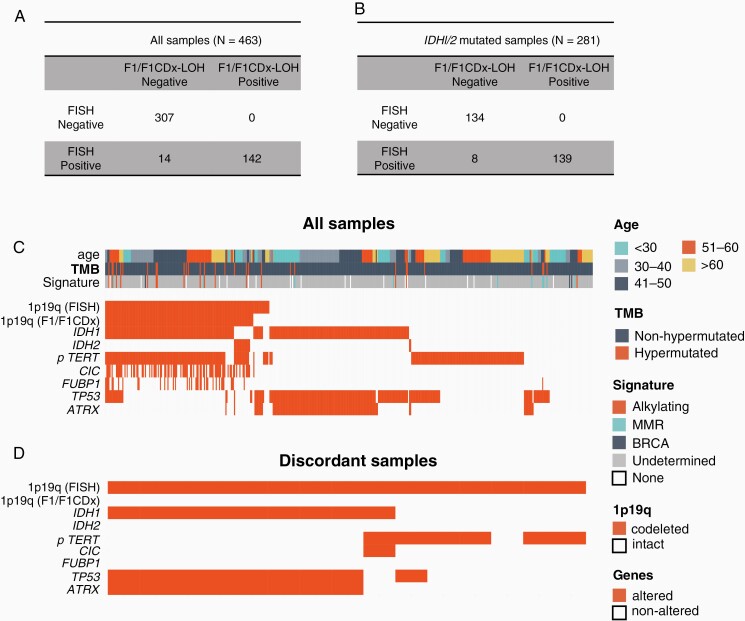
(A) Table showing the concordance results for all samples regardless of their *IDH1/2* mutation status. (B) Table showing the concordance results for *IDH1/2*-mutated samples. (C) Tile plot showing the distribution of alterations, 1p19q codeletion, age, TMB, and mutational signatures across samples used for validation. (D) Tile plot showing the distribution of alterations and 1p19q codeletion among discordant samples. F1, FoundationOne testing; F1CDx, FoundationOne CDx testing; LOH, loss of heterozygosity; TMB, tumor mutational burden; FISH, fluorescence in-situ hybridization.

Copy number and LOH status were determined for 1p and 19q in all 463 glioma samples, as illustrated in an example in Figure 2A. A sample was considered computationally 1p19q codeleted if >50% of both the 1p and 19q arms were monoallelic, i.e. lost heterozygosity in 1p and 19q (Figure 2B). We compared results from our next-generation sequencing algorithm for whole arm 1p19q codeletion to those from FISH, and concordance was assessed (Figure 3A). For all samples regardless of their *IDH1/2* status (N = 463), we observed a concordance of 96.7% (449/463, 95% CI: 95.0%–98.3%), a PPV of 100% (142/142, 95% CI: 97.4%–100%) and a PPA of 91.0% (142/156, 95% CI: 85.4%–95.0%). Samples positive for the whole arm 1p19q codeletion had a median tumor purity of 50% [range: 20%–90%], whereas samples negative for the whole arm 1p19q codeletion had a median tumor purity of 40% [20%–90%].

### Analysis of Whole Arm 1p19q Codeletion Status in IDH1/2 Mutated Gliomas

Given the accuracy of this caller across all samples, we investigated the 1p19q codeletion status specifically across *IDH1/2*-mutated glioma samples and compared our calls to FISH results (Figure 3B). For *IDH1/2*-mutated samples (N = 281), we observed a concordance of 97.2% (273/281), a PPV of 100% (139/139), and a PPA of 94.6% (139/147). The median age at testing of *IDH1/2*-mutated 1p19q-codeleted was 45 years and 36 years for *IDH1/2*-mutated 1p19q-non-codeleted samples.

### Analysis of Discordant Samples

The genomic profile of all samples used in this study is outlined in Figure 3C. We specifically noted 14 discordant samples, that were called positive for codeletion by FISH and negative by our NGS-based algorithm (Figure 3D). We saw no evidence to indicate that tumor purity impacted concordance. Six discordant samples were all negative for mutations involving *IDH1/2, CIC,* and *FUBP1* which would be uncharacteristic of oligodendroglial lineage tumors harboring a true whole arm 1p19q codeletion (Table S1). Eight discordant samples were *IDH1* mutant. Manual review of the copy number data for these samples revealed that six cases harbored partial or complete loss of one arm. Furthermore, these samples harbored co-occurring alterations involving *TP53* and *ATRX*, a genomic profile characteristic of astrocytic lineage adult diffuse gliomas. These findings suggest that the 14 discordant samples reported as 1p19q codeleted by FISH are not true oligodendrogliomas.

### Retrospective Analysis of 8127 Gliomas Assessing Histologic Diagnosis and F1/F1CDx-Derived Whole Arm 1p19q Codeletion Status

**Figure 4.**
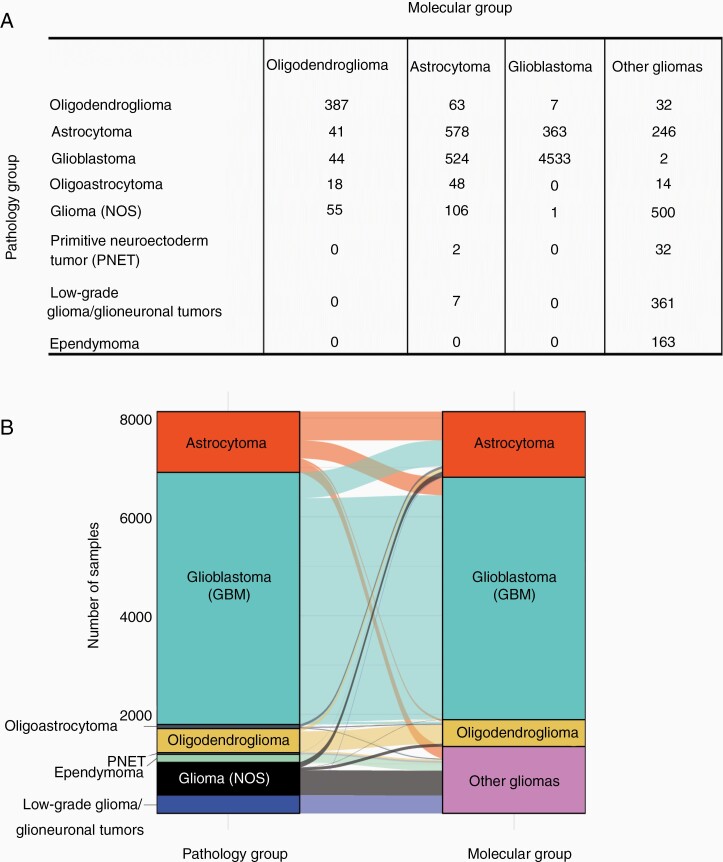
(A) Matrix showing the number of samples classified under each group by pathology (rows) vs. by molecular classification (columns). (B) An alluvial plot showing how samples are distributed across the different pathological groups vs. molecular classification.

To determine how this molecular classification can assist in a more accurate diagnosis, we analyzed 8127 glioma samples (sequenced by F1 or F1CDx assays) and binned them into molecular subgroups based on *IDH1/2* mutational status and codeletion of chromosomes 1p and 19q, as determined by F1/F1CDx (Figure 4). A total of 545 samples were classified as oligodendrogliomas, harboring *IDH1/2* mutations and 1p19q codeletion. Only 71% (387/545) were originally diagnosed as oligodendrogliomas (per the pathology report), with 29% (158/545) diagnosed as other gliomas including astrocytoma (41), oligoastrocytoma (18), GBM (44), and NOS (55) where a lineage was not specified.

We also used this approach to molecularly reclassify 80 oligoastrocytomas (as diagnosed in the pathology report), since these “mixed” lineage gliomas have lost clinical distinction, with NCCN guidelines suggesting patients be reclassified as either oligodendroglioma or astrocytoma using molecular data.^25^ Overall, 23% (18/80) were reclassified as oligodendrogliomas and 60% (48/80) were reclassified as astrocytomas. The remaining 18% (14/80) were reclassified as glioma (NOS) given their *IDH1/2* WT, *CIC* WT, and *FUBP1* WT status.

### F1/F1CDx-Derived 1p19q Codeletion is Associated with Improved Overall Survival in Glioma Patients

**Figure 5.**
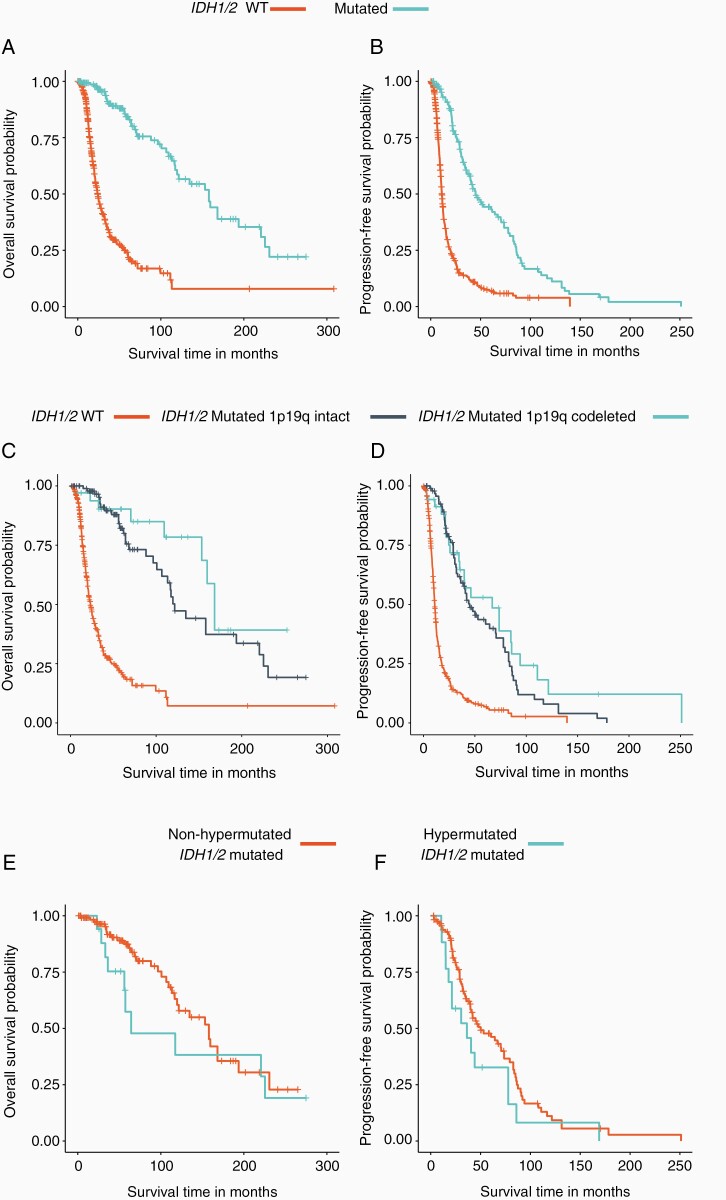
Kaplan Meier plot showing the overall survival (A) and progression-free survival (B) of *IDH1/2* WT vs. mutated samples. The overall survival and progression-free survival of *IDH1/2* WT vs. *IDH1/2* mutated 1p19q codeleted vs. *IDH1/2* mutated 1p19q intact are shown in (C) and (D). The overall survival and progression-free survival of *IDH1/2* mutated hypermutated vs. *IDH1/2* mutated non-hypermutated are shown in (E) and (F).

Next, we assessed the clinical outcomes in 519 patients samples seen at UCLA and assayed by F1 or F1CDx, where 37 patients were *IDH1/2* mutated 1p19q codeleted by F1/F1CDx, 99 patients were *IDH1/2* mutated 1p19q intact, and 383 were *IDH1/2* WT. The whole arm 1p19q codeletion status was determined from next-generation sequencing by F1 or F1CDx. Our analysis demonstrated a statistically significant survival benefit for *IDH1/2* mutated samples when compared to the *IDH1/2* WT cohort, regardless of the 1p19q codeletion status (Figure 5A). Median OS for *IDH1/2* mutated patients was 158 months (95% CI: 117–220) compared with 24 months (95% CI: 21–28) for *IDH1/2* WT patients (HR: 0.16; 95% CI: 0.11–0.23; *P* < .0001; Figure 5A). Median PFS for *IDH1/2* mutated patients was 45 months (95% CI: 40–70) compared with 11 months (95% CI: 11–12) for *IDH1/2* WT patients (HR: 0.28; 95% CI: 0.22–0.35; *P* = .002; Figure 5B).

Also, OS and PFS for tumors were assessed according to the F1/F1CDx-derived 1p19q codeletion status for *IDH1/2* mutated samples. Patients with *IDH1/2* mutated 1p19q codeleted gliomas had longer median OS (cyan, 168.2 months; 95% CI: 153.0–NA) than those with *IDH1/2* mutations without 1p19q codeletion (navy blue, 121.6 months; 95% CI: 107.0–225.0; HR: 0.42; 95% CI: 0.20–0.90; *P* < .05, Figure 5C). PFS was not statistically different between patients with *IDH1/2* mutated 1p19q codeleted gliomas (cyan, 66.9 months; 95% CI: 35.2–110.9) vs. those with *IDH1/2* mutations without 1p19q codeletion (navy blue, 44.9 months; 95% CI: 36.3–70.7, Figure 5D). Finally, we found that OS and PFS were not significantly different between patients with hypermutated *IDH1/2*-mutated tumors vs. non-hypermutated *IDH1/2*-mutated tumors, despite a trend seen towards a worse overall survival for hypermutated samples (Figure 5E and 5F). Furthermore, the overall survival and progression-free survival of discordant samples, reported as positive for 1p19q codeletion by FISH and negative by F1/F1CDx, cluster closest with the *IDH1/2* WT samples (Supplementary Figure S1A and S1B).

## Discussion

Gliomas represent a spectrum of tumors with varying lineages, histologic grades, clinical courses, and prognosis. Based on current WHO guidelines, the distinction between oligodendrogliomas and diffuse astrocytomas necessitates the detection of several molecular markers, primarily the *IDH1/2* mutation status and whole arm 1p19q codeletion status.^4^

In this study, we investigated the feasibility and accuracy of detecting the whole arm 1p19q codeletion status through comprehensive genomic profiling, instead of the traditional FISH testing. Our results showed that the computationally derived whole arm 1p19q codeletion status was highly concordant with FISH results. This was true when we assessed all glioma samples and when we restricted the analysis to only include *IDH1/2* mutated samples. In general, assessment and reporting of the codeletion status should be reserved for cases with *IDH1/2* mutations, since it has been shown that 1p19q codeletion status has no impact on the survival of *IDH1/2* WT tumors, such as glioblastomas.^26^ We also detected 1p19q codeletions in over 60% of hypermutated glioma samples, showing that hypermutations do not affect our ability to call 1p19q codeletions from F1/F1CDx.

Additionally, our work highlights the importance of F1/F1CDx testing for accurate diagnosis of gliomas. A genomic assay that detects 1p19q in addition to alterations within *IDH1/2*, *TP53*, *ATRX*, *TERT*, *TP53*, *CIC*, *FUBP1*, among others provides the complete picture. In our dataset, only 71% of *IDH1/2* mutated 1p19q codeleted samples were originally classified as oligodendrogliomas. This analysis may overstate the misdiagnosis rate since some samples have not finished the pathological workup when submitted; however, it does highlight the need for testing for relevant genomic alterations to confirm the diagnosis. Previous studies have suggested that oligoastrocytomas (OAs) showed genetic subsets characterized by either oligodendroglioma-like alterations (1p and/or 19q loss, 52% and 70% of OAs), or astrocytoma-like alterations (*TP53* mutations, 32%).^27^ Other studies have indicated gliomas previously characterized as OAs contain subsets driven by mutations to the *TERT* promoter region.^28^ Given that OAs are no longer a recognized diagnostic entity, identifying the molecular subpopulations that exist can inform the practical reclassification of this group.

Importantly, patients with F1/F1CDx-derived whole arm 1p19q codeletion showed increased overall survival compared to non-codeleted counterparts. Specifically, patients with *IDH1/2* mutations and whole arm 1p19q codeletion had better overall survival than those with *IDH1/2* mutations but no codeletion. This is in line with findings from multiple groups, showing that patients with FISH-derived 1p19q codeletion have better survival outcomes compared to patients with non-codeleted tumors.^9–18^ Furthermore, 1p19q codeletion is associated with improved temozolomide (TMZ) sensitivity, and the use of *IDH1/2* inhibitors is being investigated in clinical trials.^29,30^

Because FISH targets a single locus, positive results may indicate whole or partial arm deletion. The benefit of a comprehensive genomic profiling approach is that it can distinguish between whole and partial arm deletion. This distinction is important given the association of whole arm 1p19q codeletion with improved survival.^22^ The frequency of partial 1p19q codeletion is estimated to be at 3.6%^31^ and in our study, 6 out of the 14 total discordances were likely due to partial arm deletions, leading to a potential misdiagnosis of these cases. Our results suggest that F1 or F1CDx testing is a reliable substitute for FISH to detect the 1p19q codeletion status given its ability to distinguish partial vs. whole arm loss, in addition to describing many of the genomic alterations relevant for the molecular profiling and diagnosis of gliomas.

## Supplementary Material

vdab017_suppl_Supplementary_Material

## Notes

**Authorship Statement.** R.S., D.C.P., G.M.F., J.H., S.H.R., and L.A.A. designed the study, wrote the manuscript and developed and/or performed analyses. M.C., N.D., J.J. and B.M.A. aided in interpreting the results. T.C., W.H.Y., L.M.L., P.L.N., M.J., and A.L. provided clinical outcomes data. All authors discussed the results and contributed to the final manuscript. Portions of this work were presented at a poster discussion at the Society for Neuro-Oncology meeting, 2020.

## References

[1] Claes A , Idema AJ, Wesseling P. Diffuse glioma growth: a guerilla war. Acta Neuropathol. 2007;114(5):443–458. 17805551 10.1007/s00401-007-0293-7 PMC2039798

[2] Louis DN . Molecular pathology of malignant gliomas. Annu Rev Pathol. 2006;1:97–117. 18039109 10.1146/annurev.pathol.1.110304.100043

[3] Ostrom QT , Cioffi G, Gittleman H, et al CBTRUS statistical report: primary brain and other central nervous system tumors diagnosed in the United States in 2012–2016. Neuro Oncol. 2019;21(5):v1–v100. 31675094 10.1093/neuonc/noz150 PMC6823730

[4] Louis DN , Perry A, Reifenberger G, et al. The 2016 world health organization classification of tumors of the central nervous system: a summary. Acta Neuropathol. 2016;131(6):803–820. 27157931 10.1007/s00401-016-1545-1

[5] Yan H , Parsons DW, Jin G, et al. IDH1 and IDH2 mutations in gliomas. N Engl J Med. 2009;360(8):765–773. 19228619 10.1056/NEJMoa0808710 PMC2820383

[6] Reuss DE , Sahm F, Schrimpf D, et al ATRX and IDH1-R132H immunohistochemistry with subsequent copy number analysis and IDH sequencing as a basis for an “integrated” diagnostic approach for adult astrocytoma, oligodendroglioma and glioblastoma. Acta Neuropathol. 2015;129(1):133–146. 25427834 10.1007/s00401-014-1370-3

[7] Banan R , Hartmann C. The new WHO 2016 classification of brain tumors—what neurosurgeons need to know. Acta Neurochir (Wien). 2017;159(3):403–418. 28093610 10.1007/s00701-016-3062-3

[8] Brat DJ , Verhaak RGW, Aldape KD, et al Comprehensive, integrative genomic analysis of diffuse lower-grade gliomas. N Engl J Med. 2015;372(26):2481–2498. 26061751 10.1056/NEJMoa1402121 PMC4530011

[9] Cairncross G , Berkey B, Shaw E, et al Phase III trial of chemotherapy plus radiotherapy compared with radiotherapy alone for pure and mixed anaplastic oligodendroglioma: intergroup radiation therapy oncology group trial 9402. J Clin Oncol. 2006;24(18):2707–2714. 16782910 10.1200/JCO.2005.04.3414

[10] Cairncross JG , Ueki K, Zlatescu MC, et al. Specific genetic predictors of chemotherapeutic response and survival in patients with anaplastic oligodendrogliomas. J Natl Cancer Inst. 1998;90(19):1473–1479. 9776413 10.1093/jnci/90.19.1473

[11] Giannini C , Burger PC, Berkey BA, et al. Anaplastic oligodendroglial tumors: refining the correlation among histopathology, 1p 19q deletion and clinical outcome in intergroup radiation therapy oncology group trial 9402. Brain Pathol. 2008;18(3):360–369. 18371182 10.1111/j.1750-3639.2008.00129.x PMC8095535

[12] McLendon RE , Herndon JE 2nd, West B, et al. Survival analysis of presumptive prognostic markers among oligodendrogliomas. Cancer. 2005;104(8):1693–1699. 16116609 10.1002/cncr.21362

[13] Smith JS , Perry A, Borell TJ, et al. Alterations of chromosome arms 1p and 19q as predictors of survival in oligodendrogliomas, astrocytomas, and mixed oligoastrocytomas. J Clin Oncol. 2000;18(3):636–645. 10653879 10.1200/JCO.2000.18.3.636

[14] Erdem-Eraslan L , Gravendeel LA, de Rooi J, et al. Intrinsic molecular subtypes of glioma are prognostic and predict benefit from adjuvant procarbazine, lomustine, and vincristine chemotherapy in combination with other prognostic factors in anaplastic oligodendroglial brain tumors: a report from EORTC study 26951. J Clin Oncol. 2013;31(3): 328–336. 23269986 10.1200/JCO.2012.44.1444 PMC3732011

[15] Wiens AL , Cheng L, Bertsch EC, Johnson KA, Zhang S, Hattab EM. Polysomy of chromosomes 1 and/or 19 is common and associated with less favorable clinical outcome in oligodendrogliomas: fluorescent in situ hybridization analysis of 84 consecutive cases. J Neuropathol Exp Neurol. 2012;71(7):618–624. 22710961 10.1097/NEN.0b013e31825b5f7a

[16] Olar A , Sulman EP. Molecular markers in low-grade glioma-toward tumor reclassification. Semin Radiat Oncol. 2015;25(3):155–163. 26050585 10.1016/j.semradonc.2015.02.006 PMC4500036

[17] Labussière M , Idbaih A, Wang XW, et al. All the 1p19q codeleted gliomas are mutated on IDH1 or IDH2. Neurology. 2010;74(23):1886–1890. 20427748 10.1212/WNL.0b013e3181e1cf3a

[18] Jenkins RB , Blair H, Ballman KV, et al. A t(1;19)(q10;p10) mediates the combined deletions of 1p and 19q and predicts a better prognosis of patients with oligodendroglioma. Cancer Res. 2006;66(20): 9852–9861. 17047046 10.1158/0008-5472.CAN-06-1796

[19] Leeper HE , Caron AA, Decker PA, Jenkins RB, Lachance DH, Giannini C. IDH mutation, 1p19q codeletion and ATRX loss in WHO grade II gliomas. Oncotarget. 2015;6(30):30295–30305. 26210286 10.18632/oncotarget.4497 PMC4745799

[20] Cryan JB , Haidar S, Ramkissoon LA, et al. Clinical multiplexed exome sequencing distinguishes adult oligodendroglial neoplasms from astrocytic and mixed lineage gliomas. Oncotarget. 2014;5(18):8083–8092. 25257301 10.18632/oncotarget.2342 PMC4226668

[21] Pinkham MB , Telford N, Whitfield GA, Colaco RJ, O’Neill F, McBain CA. FISHing tips: what every clinician should know about 1p19q analysis in gliomas using fluorescence in situ hybridisation. Clin Oncol (R Coll Radiol). 2015;27(8):445–453. 25971646 10.1016/j.clon.2015.04.008

[22] Vogazianou AP , Chan R, Bäcklund LM, et al. Distinct patterns of 1p and 19q alterations identify subtypes of human gliomas that have different prognoses. Neuro Oncol. 2010;12(7):664–678. 20164239 10.1093/neuonc/nop075 PMC2940668

[23] Touat M , Li YY, Boynton AN, et al. Mechanisms and therapeutic implications of hypermutation in gliomas. Nature. 2020;580(7804):517–523. 32322066 10.1038/s41586-020-2209-9 PMC8235024

[24] Sun JX , He Y, Sanford E, et al. A computational approach to distinguish somatic vs. germline origin of genomic alterations from deep sequencing of cancer specimens without a matched normal. Plos Comput Biol. 2018;14(2):e1005965. 29415044 10.1371/journal.pcbi.1005965 PMC5832436

[25] Nabors LB , Portnow J, Ammirati M, et al Central nervous system cancers, version 1.2017 featured updates to the NCCN guidelines. JNCCN J Natl Compr Cancer Netw. 2017;15(11):1331–1345. 10.6004/jnccn.2017.0166 29118226

[26] Clark KH , Villano JL, Nikiforova MN, Hamilton RL, Horbinski C. 1p/19q testing has no significance in the workup of glioblastomas. Neuropathol Appl Neurobiol. 2013;39(6):706–717. 23363074 10.1111/nan.12031 PMC4095883

[27] Maintz D , Fiedler K, Koopmann J, et al. Molecular genetic evidence for subtypes of oligoastrocytomas. J Neuropathol Exp Neurol. 1997;56(10):1098–1104. 9329453 10.1097/00005072-199710000-00003

[28] Chan AK , Yao Y, Zhang Z, et al. TERT promoter mutations contribute to subset prognostication of lower-grade gliomas. Mod Pathol. 2015;28(2):177–186. 25081751 10.1038/modpathol.2014.94

[29] Golub D , Iyengar N, Dogra S, et al Mutant isocitrate dehydrogenase inhibitors as targeted cancer therapeutics. Front Oncol. 2019;9(MAY):1–25. 10.3389/fonc.2019.00417 PMC6534082 31165048

[30] Staedtke V , Dzaye O, Holdhoff M. Actionable molecular biomarkers in primary brain tumors. Trends Cancer. 2016;2(7):338–349. 28603776 10.1016/j.trecan.2016.06.003 PMC5461965

[31] Ball MK , Kollmeyer TM, Praska CE, et al. Frequency of false-positive FISH 1p/19q codeletion in adult diffuse astrocytic gliomas. Neurooncol Adv. 2020;2(1):vdaa109. 33205043 10.1093/noajnl/vdaa109 PMC7654379

