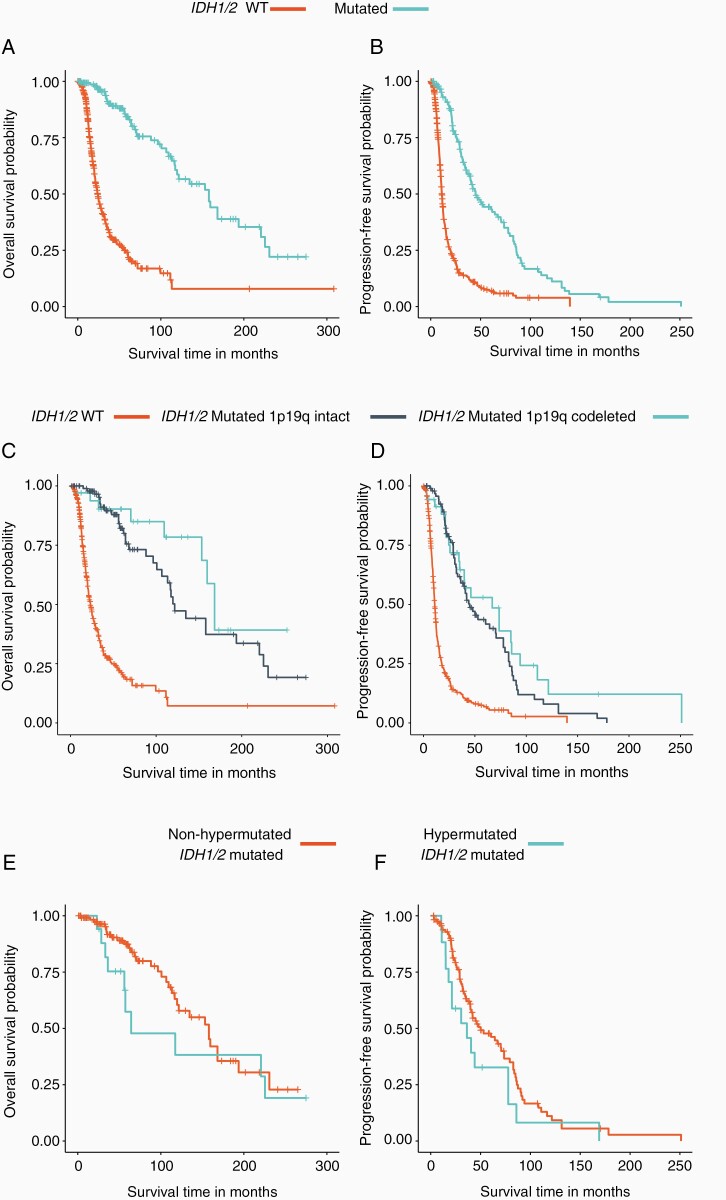# Erratum to: FoundationOne CDx testing accurately determines whole arm 1p19q codeletion status in gliomas

**DOI:** 10.1093/noajnl/vdab059

**Published:** 2021-06-22

**Authors:** Radwa Sharaf, Dean C Pavlick, Garrett M Frampton, Maureen Cooper, Jacqueline Jenkins, Natalie Danziger, James Haberberger, Brian M Alexander, Timothy Cloughesy, William H Yong, Linda M Liau, Phioanh L Nghiemphu, Matthew Ji, Albert Lai, Shakti H Ramkissoon, Lee A Albacker

In the originally published version of the manuscript, there was an error in one of the figures. Figure 5 Panel A showed the wrong curves. The corrected figure is shown here: 

This error has been corrected online.