# Supplementary material for: FoundationOne CDx testing accurately determines whole arm 1p19q codeletion status in gliomas
Source: Neurooncol Adv. 2021 Feb 4;3(1):vdab017. doi: 10.1093/noajnl/vdab017 (PMC7986056; doi:10.1093/noajnl/vdab017)
Supplement: vdab017_suppl_Supplementary_Material [file vdab017_suppl_supplementary_material.docx]

**Table S1. Overview of discordant samples**

| **Discordant sample ID** | **1p19q codeletion status by NGS** | **1p19q codeletion status by FISH** | ***IDH1/2* alteration** | ***TP53* alteration** | ***ATRX* alteration** | **Co-occurring genetic alterations** |
| --- | --- | --- | --- | --- | --- | --- |
| 1 | Intact | Codeleted | R132H | E258K, R273C | splice site 4809+2T>C | *TET2* K1827fs*6, *HGF* R134H  Gene amplifications: *CDK4, CCND2, GLI1, MYCN, FGF23, FGF6* |
| 2 | Intact | Codeleted | R132H | H178fs*71 | R937fs*5 | *GOPC-ROS1* fusion, *CDKN2A/B* homo del |
| 3 | Intact | Codeleted | R132H | R175H | K1052fs*61 | *RAF1* rearrangement intron 7, *CDKN2A/B* homo del |
| 4 | Intact | Codeleted | R132H | Y236C | S827fs*3 | none |
| 5 | Intact | Codeleted | R132H | R273C | D699fs*2 | *CDKN2A/B* homo del |
| 6 | Intact | Codeleted | R132H | R273C | E1972fs*15 | *NOTCH1* T970I |
| 7 | Intact | Codeleted | R132H | R175H | K1018fs*3 | *RAD21* homo del (ex2-9)  Gene amplifications: *CCND3*, *IGF1R* |
| 8 | Intact | Codeleted | R132H | Y220* | S460fs*9 | *AKT1* E17K, *BRCA1* E907K, *DNMT3A* A741V, *EGFR* A839T, *ERBB4* G802D, *MAP2K1* E203K, *BCL2* E165K, *AXL* P238L, *BCL2L2* A159V, *BCORL1* splice site, *CDKN1B* P26fs*99, *CREBBP* R1664C, *KDM6A* splice site, *LZTR1* W54*, *MLL2* R5021*, *MSH6* R361H, *PBRM1* L255F, *RB1* splice site |
| 9 | Intact | Codeleted | WT | R158H | WT | *PTEN* homo del ex1, *RB1* I441fs*22, homo del ex2-17, *TERT* -124C>T |
| 10 | Intact | Codeleted | WT | WT | WT | *SPTA1* R468H, *TERT* -124C>T, *MET* amplification,  *CDKN2A/B/C* homo del |
| 11 | Intact | Codeleted | WT | WT | WT | *PTCH1* D879N, *MYD88* L265P, *TERT* -124C>T, *CDKN2A/B/C* homo del  Gene amplifications: *KRAS, MDM4, PIK3C2B* |
| 12 | Intact | Codeleted | WT | WT | WT | *NRAS* amplification & G12R, *PIK3R1* K593fs*8,  *CDKN2A/B* homo del |
| 13 | Intact | Codeleted | WT | WT | WT | *TERT* -124C>T, *FANCA* R880fs*9  Gene amplifications: *CDK4, KDR, KIT, PDGFRA, HGF, MDM2, GLI1* |
| 14 | Intact | Codeleted | WT | WT | WT | *TERT* -124C>T, *TSC1* Q794*, *PTEN* homo del ex 1-5, *CDKN2A/B* homo del |

**Figure S1.** Kaplan Meier plot showing the overall survival (A) and progression-free survival (B) of discordant samples vs. *IDH1/2* WT and mutated samples.
